# Development and content validation of the Pediatric Oral Medicines Acceptability Questionnaires (P-OMAQ): patient-reported and caregiver-reported outcome measures

**DOI:** 10.1186/s41687-020-00246-1

**Published:** 2020-10-01

**Authors:** Diane M. Turner-Bowker, Kristina An Haack, Meaghan Krohe, Andrew Yaworsky, Norma Vivas, Masami Kelly, Godhuli Chatterjee, Emily Chaston, Erin Mann, Matthew Reaney

**Affiliations:** 1 Adelphi Values, 290 Congress Street, 7th Floor, Boston, MA 02210 USA; 2 Sanofi, 1 Avenue Pierre Brossolette, 91380 Chilly-Mazarin, France; 3 Sanofi Argentina S.A., Tucumán 1, 4th Floor, C1049AAA CABA, Argentina; 4 Sanofi-Synthelabo (India) Private Limited, CTS No.117-B, L&T Business Park, Saki Vihar Road, Powai, Mumbai, Maharashtra 400072 India; 5 Massachusetts Department of Public Health, Jamaica Plain, MA USA; 6 Sanofi UK, 1 Onslow Street, Guildford, GU1 4YS UK

**Keywords:** Acceptability, Pediatric oral medicines, Oral formulations, Questionnaires, Clinical outcome assessments, Caregivers, Systematic literature review, Cognitive debriefing, Concept elicitation

## Abstract

**Background:**

Evolving regulatory guidelines recommend routine assessment of the acceptability of pediatric oral medicines throughout clinical development processes. However, such assessment is problematic owing to a lack of standard methods or criteria that define acceptability for children and their caregivers. This research aimed to identify the attributes of acceptability for targeted oral formulation types that are important to children, and to develop content-valid patient- and caregiver-reported outcome acceptability measures for use in the context of clinical drug development.

**Methods:**

A concept-focused literature review and two advisory panel meetings involving researchers, clinicians, and measurement scientists were conducted to identify acceptability attributes that may be relevant to children taking targeted oral medicine formulations. The Pediatric Oral Medicines Acceptability Questionnaires (P-OMAQs), including patient (P-OMAQ-P) and caregiver (P-OMAQ-C) versions, were drafted to assess these attributes. Qualitative concept elicitation (CE) and cognitive debriefing (CD) patient and caregiver interviews were conducted to confirm key acceptability attribute concepts for measurement and to evaluate patient and caregiver ability to understand and respond to the questions.

**Results:**

A full-text review of 40 articles identified 24 acceptability attributes that were categorized into 10 overarching domains and organized into a preliminary conceptual model. Feedback from the advisory panel refined the preliminary model. In total, 14 attributes were reported during the CE phase of the interviews (*n* = 23 pediatric patients, *n* = 13 caregivers); six attributes were included in the final model. The draft P-OMAQ was refined over four waves of CD interviews (*n* = 31 pediatric patients, *n* = 48 caregivers). The final version of the P-OMAQ-P is a 12-item questionnaire designed for young people aged 8–17 years. The P-OMAQ-C is a 19-item questionnaire designed for adult caregivers of young people aged 6 months to 17 years. There are two versions of each questionnaire: one with a 24-h recall period and one with a 7-day recall period. All items are answered on a 5-point numerical rating scale.

**Conclusions:**

This research supports the content validity of the patient and caregiver versions of the P-OMAQ. Both questionnaires appropriately assess the acceptability of oral medicine formulations from the perspective of pediatric patients and their caregivers.

## Background

Medicine acceptability is defined as “a multi-faceted construct that reflects the extent to which people delivering or receiving a healthcare intervention consider it to be appropriate, based on anticipated or experienced cognitive and emotional responses to the intervention” [1]. Medicine acceptability to patients may have a substantial impact on adherence and, subsequently, on the overall efficacy and safety of a medicine [2–4]. This can pose a problem in pediatric patients; for example, an oral drug may be difficult for a child to chew or swallow; it may require administration by a parent or caregiver; or it may simply taste unappealing to the child, leading to difficulty in administration or a lack of adherence [5]. The need to establish a regulatory framework for the assessment of acceptability of pediatric medicines has gathered momentum in the past decade, following several regulatory changes, including those issued by the European Medicines Agency (EMA) and the World Health Organization [2, 6, 7]. In the 2013 *Guideline on pharmaceutical development of medicines for pediatric use*, the EMA highlighted the need to consider medicine acceptability to the patient throughout the pharmaceutical and clinical development of pediatric medicines, including studying acceptability “in children themselves as part of a clinical study involving the proposed medicinal product” [2]. Currently, the development of pediatric formulation types typically lags behind the adult formulation process, with the first opportunity for pediatric population taste assessment occurring in line with phase 2b (early-stage efficacy testing, prior to phase 3 initiation) in adult dosage development [8]. For acceptability evaluation to be as informative as possible, assessment should take place throughout clinical development (e.g. during clinical trials, pharmacological studies, and efficacy and safety studies), because evaluation of acceptability preregistration or post-marketing may support only modification of product labeling or dosing instructions [7]. Such a comprehensive assessment is integral to a full understanding of the impact of acceptability on adherence and therefore the overall effectiveness and safety of pediatric medicines [2].

In adult populations, a theoretical framework of acceptability (TFA) has been devised, consisting of seven component constructs: affective attitude, burden, perceived effectiveness, ethicality, intervention coherence, opportunity costs, and self-efficacy [1]. However, no parallel TFA exists for pediatric populations. A recent review of acceptability research in pediatrics found that there is limited evidence on how certain attributes affect the acceptability of pediatric oral medicines (such as the shape and dimensions of tablets and capsules, swallowable volumes of liquids and multiparticulates, and the size and taste of orodispersible tablets, lozenges, and chewable tablets) [9]. A mapping tool has been developed to evaluate medicine acceptability in pediatric populations, utilizing objective measures from medicine use assessments [10, 11]. According to this tool, the acceptability profiles of pediatric medicines can be divided into four clusters: “well accepted”, “accepted”, “poorly accepted”, and “not accepted”, based on the administration time, the patient’s reaction (positive/neutral/negative), and the administration method. Although this standardized method allows an evaluation of the acceptability profile, it is limited by the availability of fully validated tools for assessing pediatric medicine use and does not consider the specific attributes of acceptability that are important and relevant to pediatric patients or their caregivers.

Medicine acceptability in a pediatric population can be defined as “the overall ability and willingness of the patient to use and its caregiver to administer the medicine as intended” [2]. Although there are several publications relating to the acceptability attributes of medicines in pediatric populations [2, 9, 12, 13], with taste/overall palatability appearing to be the most extensively studied area, there is a lack of standard methodology or criteria that define what can be considered acceptable to children and their caregivers [9, 13–18]. In addition, most acceptability studies for product registration are not published, and, in the cases of those that are, there is often very little information detailing the development and validation of the acceptability tools used by the studies’ authors. No standardized measures exist for the assessment of pediatric acceptability, and available published tools have limited content coverage of the attributes that may be relevant to oral formulations. Palatability, which appears to be the most commonly studied acceptability attribute in pediatric drug development, is measured in a variety of ways, including rank order/preference and scaling methods [14, 19, 20]. For other possible attributes, assessment is influenced by the lack of a pragmatic definition for the attribute. For example, swallowability has been defined as “everything swallowed”, “smooth swallowing”, “swallowing with a choking reflex or cough”, and “biting or chewing followed by swallowing” [21].

Consequently, acceptability is not being routinely measured as part of pediatric drug development during clinical trials [20, 22]. Clinical outcome assessments (COAs) are needed to capture patient and caregiver perspectives on pediatric medicine acceptability. Specifically, there is a need to: (1) define the important and relevant attributes of acceptability from the perspective of pediatric patients and their caregivers, and (2) develop valid, reliable, sensitive, and interpretable patient- and caregiver-reported outcome measures to evaluate these attributes of medicine acceptability in the context of clinical drug development. This includes systematically testing such measures in the relevant age groups and formulation types.

The purpose of this study was to develop content-valid COAs to measure the acceptability of targeted oral medicine formulations to pediatric patients and their caregivers. The targeted oral formulation types included: tablets and mini-tablets, oral liquids or sprays, and powders or granules for reconstitution. Although other oral formulation types exist, this research aimed to develop a single standardized COA measurement strategy for as many oral formulations as possible (i.e. a COA with content relevance across the most common oral formulation types). Ethics approval was secured from the New England Independent Review Board before initiating the study on July 18, 2017.

## Methods

A content-valid COA tool measures attributes that are important and relevant to the target population and is constructed in such a way that respondents can understand and respond to the questions [23, 24]. The steps for the development of patient- and caregiver-reported acceptability measures were as follows: (1) construction of a preliminary conceptual model of acceptability attributes for targeted oral formulation types, based on a concept-focused literature review; (2) a meeting of an advisory panel of researchers, clinicians, and measurement scientists to identify preliminary acceptability attributes and refine the preliminary conceptual model; (3) construction of the draft Pediatric Oral Medicines Acceptability Questionnaires (P-OMAQs), patient version (P-OMAQ-P), and caregiver version (P-OMAQ-C), based on a review of the literature, existing questionnaires, and advisory panel input; and (4) qualitative patient and caregiver interviews consisting of a concept elicitation (CE) phase (to identify/confirm key concepts for measurement) and a cognitive debriefing (CD) phase (to test the ability of patients and caregivers to understand and respond to the draft P-OMAQ).

### Preliminary conceptual model of acceptability

#### Conceptual literature review

A targeted concept-focused review of the empirical literature was conducted to identify, describe, and substantiate attributes of acceptability that are important and relevant to pediatric patients taking oral medicines and to their caregivers. The search was implemented in OvidSP (MEDLINE, Embase, and PsycINFO) using the search strategy shown in Table S1 (Additional file 1). Resulting abstract records were screened against inclusion/exclusion criteria (see Table S1 footnotes) using Abstrackr, a tool that facilitates the screening of citations [25], to identify publications suitable for full-text review. A gray literature search was also conducted using bibliographies of publications identified for full-text review and a targeted search of Google and Google Scholar. Data from publications identified for full-text review were extracted into tables, and acceptability attributes identified from the literature were organized in an attribute description table. Brief descriptions of each acceptability attribute were included, as reported in the literature, and differences by age group and/or oral formulation type were specified where possible. Results from the literature review were used to prepare a draft conceptual model reflecting a preliminary list of acceptability attributes to consider for measurement.

#### Advisory panel meetings

A panel of clinicians, researchers, and measurement scientists from a range of geographical regions, cultures, and healthcare systems (Argentina, France, India, Turkey, and the USA) met to: (1) identify and discuss attributes of acceptability that are important and relevant for patients taking oral formulations, from the perspective of clinical and scientific advisors; and (2) identify and describe the ways in which attributes of acceptability may differ by medicine type, age of the child, and formulation. Experts were all members of Sanofi staff and were selected based on their expertise in, and current roles related to, the conduct of patient-focused pediatric research. Experts with broad experiences administering and studying pediatric medicine administration (clinically, operationally, and in research) were included. Two panel meetings were convened (each approximately 90 min in duration) by teleconference and were facilitated by two measurement scientists. A semi-structured discussion guide was used to inform the discussion and elicit information from the experts related to attributes of acceptability. During the first panel meeting, participants were asked to share their definition of medicine acceptability; generate a list of acceptability attributes for the oral formulations of interest; review acceptability attributes identified in the literature; discuss the ways in which attributes may differ based on the formulation type and the age of the child; and refine the draft conceptual model of acceptability attributes for oral formulation types. During the second panel meeting, participants prioritized and confirmed a list of key acceptability attributes for oral formulation types from the draft conceptual model. The draft conceptual model was revised.

### Questionnaire construction

#### Concept-focused review to identify existing acceptability questionnaires

A literature review was conducted to identify existing questionnaires that may be used to measure the key attributes in the revised draft conceptual model of acceptability. Searches and data extraction were performed in a similar manner to those for the concept-focused literature review (see the section “Conceptual literature review” for methodology). Abstracts were reviewed to identify studies reporting on the development or use of questionnaires to evaluate medicine acceptability in a pediatric population. The content of the identified questionnaires was then reviewed and compared against the draft conceptual model of acceptability, to evaluate the conceptual coverage of these questionnaires.

#### Development of draft patient and caregiver acceptability questionnaires

Because no single existing questionnaire appears to measure the key attribute concepts identified through the concept-focused literature review and advisory panel discussions, new questionnaires were drafted for the assessment of the targeted oral formulation acceptability attributes. Instructions, questions, and response options were written to measure each acceptability attribute in the conceptual model.

### Establishing content validity

#### Combined CE and CD interviews with patients and caregivers

In-person or telephone-based hybrid (combined) CE and CD interviews were conducted with pediatric patients taking an oral medicine (mini-tablets/tablets, oral liquids/sprays, or powders/granules for reconstitution) and/or their caregivers. The purpose of the CE portion of the interviews was to confirm key acceptability attributes for measurement, and to finalize the draft conceptual model. The purpose of the CD portion of the interviews was to evaluate the respondents’ understanding and their ability to complete the draft patient and caregiver acceptability questionnaires.

Potential participants were identified, and consent was obtained, before screening through an external commercial recruitment agency that contracts with clinical sites. Patients were recruited from 19 private US-based pediatric practices and multispecialty clinical sites with a focus on pediatric treatment across three regions, as shown in Table S2 (Additional file 2). Recruitment initially targeted the inclusion of 36 pediatric patients (and their caregivers), aged 6 months to 17 years, currently taking an oral treatment. Targets were developed to recruit a diverse participant sample based on age and oral formulation type and were not limited by therapeutic area or to patients taking Sanofi treatments. Efforts were made to include patients being treated for acute or chronic conditions (the targeted distributions for length of time on treatment were 0–1 month, 1–6 months and > 6 months) and also those receiving prophylactic treatment (in addition to symptom treatment). The inclusion and exclusion criteria for the interviews can be found in additional information (Additional file 3). Participants (pediatric patient or caregiver) were compensated $150.00 upon completion of a 90-min interview or $100.00 upon completion of a 30-min interview.

Interviews were completed in waves. This allowed for new acceptability attributes identified in the CE portion of the interview to be added to the conceptual model, and for new P-OMAQ item(s) covering this content to be drafted and tested in the next wave. If needed, original draft items were also revised between waves and tested in subsequent waves. Three waves were initially planned for CE and CD; an additional fourth wave of CD interviews was conducted to confirm a more limited, specific set of revisions to the questionnaire among children aged 8–11 years (although caregivers reviewed content and provided feedback, they were not debriefed in depth on the questionnaire in Wave 4).

The CE interviews were conducted using a semi-structured interview guide in the following manner: the caregivers of pediatric patients aged 6 months to 5 years were interviewed to elicit their observations in relation to the child’s experience of taking an oral medication; the pediatric participants aged 6–17 years were given the option of having a caregiver present during the interview. Although caregivers were also invited to participate in these interviews, the primary interaction was with the child to gather direct input about their experience of taking an oral medication. Because the P-OMAQ-P was designed for pediatric participants aged 8 years and older, the CD portions of the interviews were conducted using a semi-structured interview guide in the following manner: children aged 8–17 years completed and were cognitively debriefed on the questionnaire; pediatric participants who were 6 or 7 years of age participated in the CE interviews but were not asked to complete the questionnaire; the caregivers of all patients were cognitively debriefed on the P-OMAQ-C.

The CE and CD components of the interviews lasted approximately 60 min and 30 min, respectively (for a total of approximately 90 min per interview). Interviews were audio-recorded and transcribed. Each transcript was then anonymized to remove any potentially identifying information.

Transcripts were coded using ATLAS.ti version 7.0 (ATLAS.ti Scientific Software Development GmbH, Berlin, Germany) to facilitate analysis and interpretation. Coding was an iterative process, in which several researchers identified transcript text where a participant described a concept or topic relevant to the study objectives. Throughout the coding process, a ‘codebook’ was used to match the text to a code that best characterizes the concept or topic. Before coding began, researchers developed two “codebooks”, one for patients and one for caregivers. A codebook is a comprehensive list of all the codes used to characterize important segments of transcript text and is used as a guide to organize the content and meaning of actual participant language. The preliminary coding scheme was developed before coding and was modified as coders analyzed the transcripts. When a new concept or topic was identified during coding, a new code was created if no existing code applied to the identified concept or topic. Using the codebook, participants’ perspectives on acceptability concepts, as well as their ability to understand and respond to the questionnaires, were classified and organized for further analysis.

To facilitate data analysis, findings tables consisting of verbatim quotes were generated for both pediatric patients and caregivers. An acceptability attribute concept tracking matrix table was also developed to summarize the attributes reported during the CE component of the interviews, and this table included additional information such as how frequently each attribute was reported and whether the attribute was reported as being important to participants.

Data from the CE component of the interviews were analyzed by calculating the number of patients who expressed a given acceptability attribute concept during the interview. This provided a concept frequency count of the total number of unique participants who stated that they had experienced the attribute concept at least once over the course of their experience with a specific oral-administered medication.

Data from the CD component of the interviews were qualitatively reviewed to describe: the ability of participants to understand the instructions, items, and response options of the questionnaires as intended; their ability to meaningfully respond to questionnaire items; and the relevance of the attribute concepts assessed by each item. Additionally, the data were used to indicate suggestions for rewording of the questionnaires, if any, as well as whether any attribute concepts were missing from them.

## Results

### Preliminary conceptual model of acceptability

#### Conceptual literature review

**Fig. 1:**
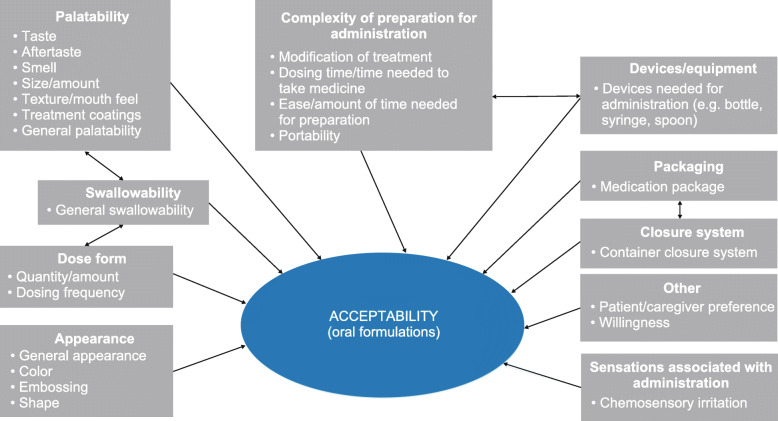
Literature-based preliminary conceptual model of oral formulation acceptability attributes

In total, 40 articles were reviewed as a part of the concept-focused literature search [2, 3, 5, 9, 10, 12, 14, 15, 17, 19, 26–55], as shown in Fig. S1 (Additional file 4). Overall, 24 acceptability attributes emerged and were categorized into 10 overarching domains: appearance; closure system; complexity of preparation (or modification) needed for administration; devices/equipment; dose form; palatability; packaging; sensations associated with administration; swallowability; and other. These results were used to form a preliminary conceptual model, as shown in Fig. 1.

#### Advisory panel meeting

**Fig. 2:**
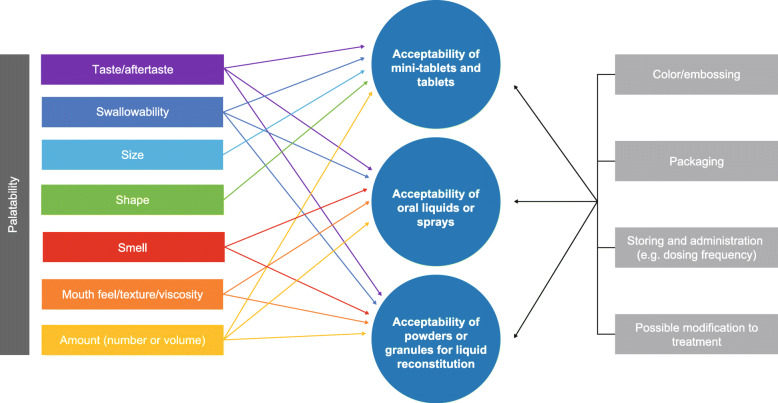
Draft conceptual model of targeted oral formulation acceptability attributes following advisory panel

Advisory panel meeting participants (*n* = 5) were presented with the preliminary conceptual model (Fig. 1) and confirmed that key acceptability attributes for oral formulations include: appearance (packaging, color), smell, size, shape, taste/aftertaste, viscosity/mouth feel/texture, quantity, swallowability, storage and administration, and possible modification to treatment (e.g. splitting tablet in half prior to administration). Participants indicated that acceptability attributes common to tablets, liquids/sprays, and powders/granules for reconstitution included: packaging, color/embossing, taste/aftertaste, quantity, storage and administration, and possible modification of treatment. Participants considered that size and shape attributes are uniquely relevant to tablets, and that smell and viscosity/mouth feel/texture attributes may be uniquely relevant to liquids/sprays and powders/granules for liquid reconstitution. Participants also noted potential attribute differences by age of the patient and treatment duration (short- vs long-term treatment). For example, participants agreed that the visual appearance of a medicine is important to children aged 5 years or younger but may not be as important for adolescents (aged 12–17 years). Participants also noted that 6–11-year-olds may prefer a medicine that does not “look” like a medicine in case they need to take the treatment while at school. Based on discussion, revisions were made to the conceptual model to reflect what were determined to be the most salient attributes related to acceptability for each oral formulation type (Fig. 2).

### Questionnaire construction

#### Literature review to identify existing acceptability questionnaires

In total, 16 articles describing nine existing questionnaires that primarily focus on assessing the acceptability of oral formulations among children (aged < 18 years) were identified [10, 17, 26, 28–30, 32, 35, 36, 40, 44, 45, 47, 49, 50, 54], as shown in Fig. S2 (Additional file 5). These 16 articles were also identified in the section “Conceptual literature review”. The acceptability attributes measured by the identified existing questionnaires included overall acceptability, palatability (defined as a single attribute combining flavor, smell, and overall liking of the medicine), and swallowability, among other more general concepts (e.g. overall perception). Taste was the most frequently measured acceptability attribute (identified in nine articles, 56%), followed by general acceptability (identified in six articles, 38%) and overall palatability and swallowability or ease of intake (identified in five articles each, 31%). A summary of the attribute concepts measured, recall periods, and question response formats (e.g. visual analog scale, numerical rating scale [NRS], and symptom checklist) covered by each questionnaire is provided in Table 1.

**Table 1:** Summary of the concepts by existing questionnaires identified and the Pediatric Oral Medicines Acceptability Questionnaires

Key attribute concepts	Medication acceptance scale ^b^ [40, 45]	Visual analog scale ^b^ [9, 26, 28, 32, 49, 50, 54]	NRS ^b^ [36]	6-point face hedonic scale ^b^ [35]	5-point face hedonic scale ^b^ [44]	4-point face hedonic scale ^b^ [17]	3-point face hedonic scale ^b^ [10]	4-point Likert scale ^b^ [10, 17, 44]	5-point Likert scale ^b^ [17, 29, 32, 40, 54]	P-OMAQ-P ^c^ (5-point NRS)	P-OMAQ-C ^c^ (5-point NRS)
**Swallowability**	**✓**	**✓**		**✓**			**✓**	**✓**		**✓**	**✓**
**Taste (before swallowing)**		**✓**	**✓**		**✓**		**✓**			**✓**	**✓**
**Aftertaste**										**✓**	**✓**
**Texture/mouthfeel**		**✓**								**✓**	**✓**
**Size/amount**										**✓**	**✓**
**Smell**					**✓**					**✓**	**✓**
**Number of times had to take**										**✓**	**✓**
Overall happiness with the medicine										✓	✓
How much the medicine helps										✓	✓
Importance to take the medicine										✓	✓
Willingness to keep taking the medicine										✓	✓
How often the child takes the medicine exactly as instructed										✓	✓
Pleasant/bothersome		✓									
Acceptability ^a^	✓	✓				✓			✓		✓
Ease of intake								✓			
Palatability		✓					✓	✓	✓		
Patient expression	✓						✓				
Ease of preparation								✓			
Overall perception									✓		

Concepts shown in bold are included in the conceptual model of acceptability of pediatric oral medicines

*NRS* Numerical Rating Scale, *P-OMAQ-C* Pediatric Oral Medicines Acceptability Questionnaires – Caregiver, *P-OMAQ-P* Pediatric Oral Medicines Acceptability Questionnaires – Patient

^a^Acceptability identified via conceptual literature review

^b^Recall period: present moment after taking

^c^Recall period: 7-day and 24-h

Although some of the key acceptability attributes identified through the concept-focused literature review and panel discussions (and reflected in Fig. 2) were included in some of the existing questionnaires, no single existing questionnaire measures all of these key attributes.

#### Development of draft P-OMAQ

Because no single existing questionnaire appears to measure the key attribute concepts identified through the concept-focused literature review and advisory panel discussions, new questionnaires were drafted for the assessment of the targeted oral formulation acceptability attributes, collectively referred to as the P-OMAQ (see Table 1). Two different versions were developed: (1) the P-OMAQ-P and (2) the P-OMAQ-C.

The P-OMAQ-P was drafted as a 13-item patient reported outcome (PRO) for pediatric respondents intended for self-reporting by patients aged 8–17 years, because evidence shows that self-reports by children aged 8 years and above frequently meet generally accepted standards for reliability on both generic and condition-specific measures [56]. For the oral medication taken now, seven items measured how happy/unhappy the child is with the following: number of times taken daily, size or amount, smell, taste, taste in the mouth after taking the medicine, how the medicine feels in the mouth, and ease of swallowing. Five items measured overall happiness with the medicine, how much the medicine helps, the importance of taking the medicine, bother, and willingness to keep taking the medicine. One item measured how often the child takes the medicine exactly as instructed.

The P-OMAQ-C was drafted as a 21-item COA for adult caregivers (aged ≥18 years) of pediatric patients aged 6 months to 17 years. Three PRO items measured, for the oral medication taken by the child now, how happy/unhappy the caregiver is with the following: number of times taken daily, size or amount, and smell. Six PRO items measured overall: acceptability of the child’s medicine to the caregiver; happiness with the child’s medicine; how much the medicine helps the child; the importance of the child taking the medicine; how much the caregiver is bothered by the child having to take the medicine; and willingness to have the child continue to use the medicine. Twelve caregiver-reported outcome items measured, for the oral medications taken by the child now, what the caregiver has directly observed (or what the child has said) about the child’s experience of taking the medicine. Of these, six items measured (based on the caregiver observation) how happy/unhappy the child is with the number of times taken daily, size or amount, smell, taste, taste in the mouth after taking the medicine, and how the medicine feels in the mouth; one item measured the caregiver’s observation of the ease of swallowing the medicine for the child; four items measured the caregiver’s perspective on overall acceptability to the child, happiness with the medicine, bother, and willingness to continue to use the medicine; and one item measured the caregiver’s perspective on how often the child takes the medicine as instructed. All the items included in the P-OMAQ-P and P-OMAQ-C used a 5-point NRS (with an 11-point NRS tested as an alternative response format). The initial recall period for both questionnaires was “now”.

### Combined CE and CD interviews with patients and caregivers

A total of 36 interviews (with both CE and CD components) were conducted across three waves. An additional wave of 12 CD-only interviews was conducted to further evaluate whether younger participants could read and understand the revised questionnaires, with a particular focus on assessing comprehension of the 24-h and 7-day recall periods. Table 2 presents demographic information for the pediatric participants and their caregivers, the oral formulation type, the health status, and the current health conditions of the pediatric participants, and Table 3 shows the distribution of participants by interview wave, age, and oral formulation type. Table S3 (Additional file 6) includes additional caregiver demographic information. Table S2 (Additional file 2) shows participant recruitment by site and interview wave for the patient and caregiver interviews.

**Table 2:** Patient and caregiver demographic information and patient health information

	Patients (*N* = 48)	Caregivers (*N* = 48)
Age, years		
Mean (SD)	9.2 (4.1)	41.6 (8.9)
Range	1.1–17.7	29.1–75.0
Sex, *n* (%)		
Female	23 (47.9)	45 (93.8)
Male	25 (52.1)	3 (6.3)
Race, *n* (%)		
Asian	1 (2.1)	2 (4.2)
Black or African American	9 (18.8)	9 (18.8)
White/Caucasian	33 (68.8)	33 (68.8)
Other	5 (10.4)	4 (8.3)
Spanish/Hispanic/Latino, *n* (%)		
Non-Spanish/Hispanic/Latino	42 (87.5)	43 (89.6)
Mexican/Mexican American/Chicano	1 (2.1)	1 (2.1)
Puerto Rican	3 (6.3)	3 (6.3)
Other	2 (4.2)	1 (2.1)
Oral formulation type, *n* (%)		
Pill (tablet or mini-tablet)	16 (33.3)	n/a
Liquid (oral spray or oral syringe)	16 (33.3)	n/a
Powder or granule that is mixed with liquid before taking	16 (33.3)	n/a
Health status ^a^, *n* (%)		
Excellent	28 (58.3%)	n/a
Very good	14 (29.2%)	n/a
Good	3 (6.3%)	n/a
Fair	3 (6.3%)	n/a
Poor	0 (0.0%)	n/a
Current health conditions, *n* (%) ^b, c^		
Allergies	9 (12.5%)	n/a
Anemia	0 (0.0%)	n/a
Arthritis	0 (0.0%)	n/a
Asthma	8 (11.1%)	n/a
Attention deficit hyperactivity disorder (ADHD)	6 (8.3%)	n/a
Attention deficit disorder (ADD)	4 (5.6%)	n/a
Autism	0 (0.0%)	n/a
Cerebral palsy	0 (0.0%)	n/a
Crohn’s disease	0 (0.0%)	n/a
Cystic fibrosis	0 (0.0%)	n/a
Diabetes	1 (1.4%)	n/a
Down syndrome	0 (0.0%)	n/a
Developmental delays	0 (0.0%)	n/a
Ear infections	8 (11.1%)	n/a
Headaches	4 (5.6%)	n/a
Hearing loss	1 (1.4%)	n/a
Heart disease	0 (0.0%)	n/a
Irritable bowel syndrome	2 (2.8%)	n/a
Intellectual disability (also known as mental retardation)	0 (0.0%)	n/a
Muscular dystrophy	0 (0.0%)	n/a
Seizures	0 (0.0%)	n/a
Sickle cell anemia	0 (0.0%)	n/a
Ulcerative colitis	0 (0.0%)	n/a
Vision loss	0 (0.0%)	n/a
Other (specified)	0 (0.0%)	n/a
Anxiety	1 (1.4%)	n/a
Constipation	7 (9.7%)	n/a
Eczema	1 (1.4%)	n/a
Eosinophilic esophagitis	1 (1.4%)	n/a
Fever/cold	4 (5.6%)	n/a
Gastritis	1 (1.4%)	n/a
Migraines	1 (1.4%)	n/a
Mitral regurgitation	1 (1.4%)	n/a
Pharyngitis	1 (1.4%)	n/a
Stomach disorder	1 (1.4%)	n/a
None	10 (13.9%)	n/a

*n/a* not applicable, *SD* standard deviation

^a^Self-reported by *n* = 22 participants aged 12–17 years; proxy-reported by *n* = 26 caregivers of participants aged 6 months to 11 years

^b^Based on responses to the question, “What health conditions do you currently have, if any? (Please choose all that apply)”

^c^Each participant could select more than one response to this question, so the total number of conditions reported will not equal the number of participants in the study

**Table 3:** Distribution of participants by wave, age, and formulation

Age	Liquid, *n*	Powders, *n*	Pills or tablets, *n*	Number of interviews, *n*
Wave 1				
6 months to 5 years ^a^	1	1	0	2
6–7 years ^b^	1	2	0	3
8–11 years ^b^	2	0	3	5
12–17 years ^c^	1^e^	0	1	2
Subtotal	5	3	4	12
Wave 2				
6 months to 5 years ^a^	2	2^d^	2	6
6–7 years ^b^	0	0	0	0
8–11 years ^b^	1	1	1	3
12–17 years ^c^	2	1	1	4
Subtotal	5	4	4	13
Wave 3				
6 months to 5 years ^a^	1	2	2	5
6–7 years ^b^	0	1	0	1
8–11 years ^b^	0	1	0	1
12–17 years ^c^	1	1	2	4
Subtotal	2	5	4	11
Wave 4 ^e^				
8–11 years ^b^	4	4	4	12
Subtotal	4	4	4	12
Total	16	16	16	48

Concept elicitation interviews were not carried out during Wave 4, and only patients aged 8–17 years and caregivers of all patients were included in the cognitive debriefing interviews

^a^Caregiver only

^b^Patient and caregiver

^c^Patient (caregivers were invited to participate); separate consent was gathered from the patient and the caregiver

^d^The patient administered her/his medicine through a nebulizer

^e^The goal of the Wave 4 interviews was to cognitively debrief the questionnaire among the youngest age group that would be asked to complete the questionnaire; therefore, only participants aged 8–11 years were asked to participate in this wave of interviews

#### Results from CE interviews

In total, 14 oral treatment acceptability attribute concepts were spontaneously reported during the CE stage of interviews (Table 4). Key concepts emerging with the highest frequency of report included taste before swallowing (reported by 82.6% of pediatric patients and 92.3% of caregivers), texture/mouth feel (reported by 82.6% of pediatric patients and 84.6% of caregivers), swallowability (reported by 78.3% of pediatric patients and 76.9% of caregivers), size/amount (reported by 73.9% of pediatric patients and 76.9% of caregivers), and aftertaste (taste after swallowing, reported by 47.8% of pediatric patients and 46.2% of caregivers). Table 4 shows the frequencies of all acceptability attributes reported by pediatric patients and caregivers and provides examples of quotations from pediatric patients and caregivers for the most frequently reported attributes.

**Table 4:** Frequency (%) of attributes and sample quotations reported during concept elicitation interviews (Waves 1–3)

Concepts (attributes) ^a,b^	Pediatric-reported, % (*n*) (*N* = 23 ^d^)	Caregiver-reported, % (*n*) (*N* = 13 ^e^)	Sample quotations by pediatric patients	Sample quotations by caregivers
Taste (before swallowing) ^a^	82.6 (19)	92.3 (12)	“I mean it tastes a little weird … It’s like sometimes it’s hard to do it all in one go just because of the taste and everything” (age 11, liquid)	“So at first she was kind of complaining that it tasted weird” (age 3, tablet)
Texture/mouth feel ^a^	82.6 (19)	84.6 (11)	“Like you accidentally eat sand” (age 12, pill)	“He complains about the grittiness of it … like if you were anybody who’s ever eaten like grit that gritty feeling that you get, that aftertaste in your mouth” (age 12, pill)
Swallowability ^a^	78.3 (18)	76.9 (10)	“It’s easy to swallow because it’s a liquid” (age 9, liquid)	“At first, um, it was harder for her to … understand how to swallow it and stuff” (age 3, tablet) “So I think that maybe if the texture was thinner and easier to swallow, um, that it probably would be better” (age 2, liquid)
Size/amount ^a^	73.9 (17)	76.9 (10)	Question: “What is the most important thing for you when you decide if you like the medicine or not?” Answer: “How it goes down and how big it is” (age 15, tablet)	“He will sometimes just tell me, you know, that he doesn’t like taking it. Or, um, that it’s just too much.” (age 5, pill)
Aftertaste (taste after swallowing) ^a^	47.8 (11)	46.2 (6)	It didn’t really taste much once you swallowed it, but then a couple seconds later, um, then it just started to taste bitter” (age 10, tablet)	“He wants to gag. So, I try to quickly give him some water … or like some apple juice so that that taste can get out of his mouth as quickly as possible” (age 4, liquid) “… he don’t like the taste of it, it leaves a nasty taste, a nasty taste on his tongue … if he don’t like it. He will throw it up … he’ll remember that taste and he’ll slap it to the floor” (age 4, pill)
Smell ^a^	43.5 (10)	53.8 (7)	Question: “And what did it smell like to you?” Answer: “Nasty … Bananas” (age 6, pill)	“… he will say, ‘I don’t like this smell.’ Or, ‘I’m not going to take this.’ And he won’t take it. So that’s one thing about him, is if it’s something that he don’t like and it’s making him sick, he doesn’t take it” (age 4, pill)
Dosing frequency ^b^	8.7 (2)	7.7 (1)	n/a	n/a
**Efficacy (helping patient to feel better)** ^**c**^	**30.4 (7)**	**30.8 (4)**	**n/a**	**n/a**
**Side effects** ^**c**^	**17.4 (4)**	**7.7 (1)**	**n/a**	**n/a**
**Preparation** ^**c**^	**8.7 (2)**	**76.9 (10)**	**n/a**	**n/a**
**Appearance/color** ^**c**^	**17.4 (4)**	**30.8 (4)**	**n/a**	**n/a**
**Overall ease of administration** ^**c**^	**4.3 (1)**	**Not reported**	**n/a**	**n/a**
**Packaging** ^**c**^	**Not reported**	**7.7 (1)**	**n/a**	**n/a**
**Portability** ^**c**^	**Not reported**	**7.7 (1)**	**n/a**	**n/a**

*n/a* not applicable

^a^Concepts listed in normal (nonbold) font were included in the questionnaire

^b^Concepts were retained despite being reported at a low frequency during the concept elicitation interviews based on patient endorsement during cognitive debriefing interviews

^c^Concepts shown in bold were identified during the literature review and advisory panel meetings but were not included in the questionnaire, as they were reported at a low frequency during the concept elicitation interviews

^d^The total number of children interviewed in concept elicitation during Waves 1–3 was *n* = 23 (caregivers of children aged 6 months to 5 years were interviewed instead of the children). Frequency reports presented here are representative of patient feedback (i.e. additional caregiver feedback that may have been provided during concept elicitation is not included here)

^e^The total number of caregivers interviewed in concept elicitation during Waves 1–3 was *n* = 13 for children aged 6 months to 5 years

**Fig. 3:**
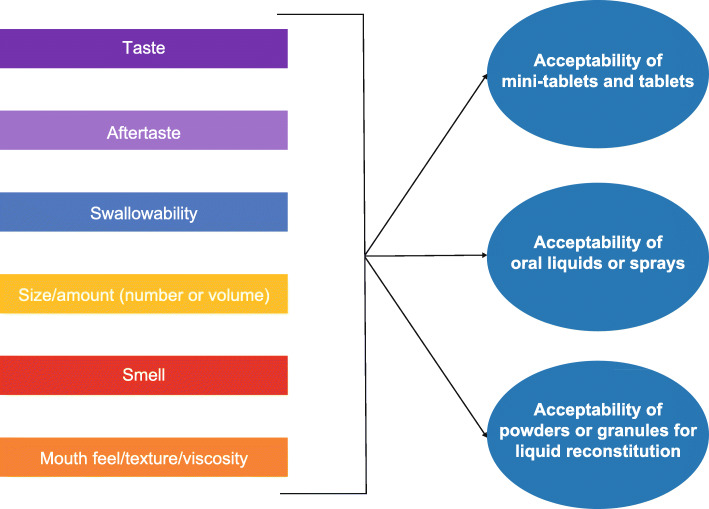
Final conceptual model of targeted oral formulation key acceptability attributes following patient and caregiver interviews

A summary of frequencies of attributes by age and oral formulation type is included in Table S4 (Additional file 7). Table 5 provides an example of the attribute concept tracking matrix used to collate information gathered from the concept-focused literature review, advisory panel meetings, and CE interviews. This information was used to develop the final version of the conceptual model. The following revisions were made to the conceptual model after the CE interviews: color/embossing, packaging, storage and administration, and possible modification to treatment were removed; taste and aftertaste were divided into separate concepts; size and amount (number or volume) were combined into a single concept; and the format of the model was revised to reflect that smell may also apply to mini-tablets or tablets. The final conceptual model, reflecting the predominant, or key, acceptability concepts identified and confirmed by patients and caregivers, is shown in Fig. 3.

**Table 5:** Example of the concept tracking matrix for attributes included in the Pediatric Oral Medicines Acceptability Questionnaires

Concept description and example quote	Frequency of participant report, *n* (%) (*N* = 36) (Pediatric: *n* = 23) (Caregiver: *n* = 13)	Reported in literature, *n* (%) (*N* = 40 articles)	Reported or confirmed by advisors
Taste (before swallowing) The way the medicine tasted when in the patient’s mouth when taking the medicine (e.g. bitter, salty, sweet) was deemed to be an important attribute across all formulation types For participants who reported an unpleasant experience with the taste of their medicine, described it as being “bitter”, having a “sourness”, and/or tasting “metallic”. Others used more general terminology, simply saying it tasted “very strong”, or had a “bad taste”. Some patients reacted negatively to “artificial” fruit flavors (e.g. “bad excuse for cherry”)	Pediatric: 19 (82.6) Caregiver: 13 (100.0)	24 (60)	Yes
Texture/mouth feel In general, participants with tablets/pills described the way the medicine feels when in their mouth as “smooth”, and they did not report any difficulties/problems associated with this attribute When the way the medicine felt in the patient’s mouth was perceived as negative or unpleasant, this attribute was described as “heavy”, “weird”, “gritty”, “chunky”, and “thick” by participants with liquid medicine. With powdered medicine, it was described as “kind of like sand”	Pediatric: 19 (82.6) Caregiver: 11 (84.6)	11 (27.5)	Yes
Swallowability Described by participants as the ability to/ease with which one can swallow the medicine In general, participants reported that they did not have issues swallowing their current medicine, with some describing the process as “easy” or “not [getting] as stuck in my throat as other pills do”	Pediatric: 19 (82.6) Caregiver: 9 (69.2)	17 (42.5)	Yes
Size/amount Size of pills/tablets was discussed within the context of the diameter or dimension of the medicine (e.g. “less than a half inch”) or a broad descriptor (e.g. “it was medium… Not a lot”) Amount of medicine was discussed within the context of the number of pills/tablets taken with each dose, or in the case of liquids the total volume taken with each dose (e.g. “a cup of it” or “a teaspoon”)	Pediatric: 17 (73.9) Caregiver: 12 (92.3)	16 (40.0)	Yes
Dosing frequency Both patients, and the caregiver, who reported this concept indicated that administering the medicine less frequently (e.g. taking medicine once per day instead of twice per day) would be preferable	Pediatric: 2 (8.7%) Caregiver: 1 (7.7%)	2 (4.9)	Yes

#### Results from CD interviews

Based on results from Waves 1–3 (in which both questionnaires were administered and debriefed in their entirety), most pediatric patients (*n* = 17, 89.4%) and caregivers (*n* = 34, 97.1%) reported that the P-OMAQ-P and P-OMAQ-C questionnaires were easy to complete. Across Waves 1–4, all participants aged 8–11 years (*n* = 21, 100.0%) were able to complete the questionnaire without assistance from their caregivers. The P-OMAQ-P and P-OMAQ-C were revised to address patient and caregiver feedback on the content coverage of the questions and any issues identified in each wave. Wave 4 interviews confirmed revisions to the instructions and recall period and overall results regarding the minimum age for self-report among the younger self-reporting participants (aged 8–11 years). Questionnaire revisions are summarized below and revisions by wave are detailed in Table 6.

**Table 6:** Revisions to the P-OMAQ-P and P-OMAQ-C questionnaires following the concept elicitation and cognitive debriefing interviews

	P-OMAQ-P	P-OMAQ-C
Wave 1	No changes	No changes
Wave 2	Recall period changed from “now” to “currently” Example: “Please think about the medicine you take by mouth CURRENTLY for (condition) and answer the questions below. Select one answer for each question.” Tested recall periods (24 h, 7 days, and 2 weeks)	
	Added item*:* “How happy are you with the *taste* of the medicine in your mouth *before swallowing*?” Removed item: “Overall, how much does the medicine *bother* you?”	Added item: “How happy is your child with the *taste* of the medicine in his/her mouth *before swallowing*?” Removed items: “Overall, how *bothered* are you by your child having to take the medicine?” and “Overall, how much does the medicine *bother* your child?”
Wave 3	Revised recall period from “currently” to test “past 24 h” AND “past 7 days” Example: “Please think about the study medication you have taken in the past 24 h/past 7 days when answering the questions. Select one answer for each question.”	
	Revised item 1 from: “How happy are you with the *number of times* you take the medicine each day?” to “How happy are you with the *number of times* you had to take the medicine?” Removed item: “How happy are you with the taste of the medicine?” and replaced with “How happy are you with the *taste* of the medicine in your mouth *before swallowing*?”	Revised item 1 to: “How happy are you with the *number of times* your child had to take the medicine?”
Wave 4	Revised items 1–7 to include recall period within the item stem: “*In the past 24 h*, how happy were you with the number of times you had to take the medicine?” “*In the past 7 days,* how happy were you with the number of times you had to take the medicine?”	Revised items 1–3 and 9–15 to include recall period within the item stem: “In the *past 24 h*, how happy were you with the *number of times* your child had to take the medicine?” “In the *past 7 days*, how happy were you with the *number of times* your child had to take the medicine?”
Example questions from the final version of the questionnaire	“In the [past 24 h/past 7 days], how happy were you with the size or amount of the medicine?” “In the [past 24 h/past 7 days], how happy were you with the smell of the medicine?” “Overall, how happy are you with the medicine?”	“In the [past 24 h/past 7 days], how happy were you with the size or amount of your child’s medicine?” “In the [past 24 h/past 7 days], how happy were you with the smell of your child’s medicine?” “Overall, how willing are you to have your child continue to use the medicine?”

*P-OMAQ-C* Pediatric Oral Medicines Acceptability Questionnaires – Caregiver, *P-OMAQ-P* Pediatric Oral Medicines Acceptability Questionnaires – Patient

##### Instructions and recall period revisions

The draft P-OMAQ-P and P-OMAQ-C used “now” as the recall period. Wave 1 results indicated that both patients and caregivers interpreted “now” inconsistently (i.e. they reported different time periods when thinking of the term “now”). In Wave 2, a revised set of instructions including “currently” in place of “now” were tested, and results were similar to Wave 1. For example, one participant defined “currently” as “whenever I took it”, whereas another participant defined “currently” as “in the last 24 hours”. Wave 3 tested two new alternative instruction sets: one with “past 24 hours” and one with “past 7 days” as the recall period, and both were appropriately interpreted by patients and caregivers during Wave 3. Because Wave 3 interviews included only one pediatric self-reporting participant aged 8–11 years (see Table 3), Wave 4 interviews were conducted with an additional 12 self-reporting pediatric participants aged 8–11 years and included the recall period both in the instruction and in the stem of each item. Results from Wave 4 confirmed patients’ understanding of the “past 24 h” and “past 7 days” recall periods in this group. Specifically, the majority of Wave 4 pediatric participants (*n* = 11, 91.7%) and all 12 caregivers (100.0%) interpreted the 24-h and 7-day recall periods from the instructions as intended. While pediatric participants interpreted the recall periods and instructions as intended, after the first eight Wave 4 interviews were completed, the findings indicated that most patients (*n* = 5, 62.5%) were not utilizing the recall period as intended. Therefore, the recall periods were added to the item stem in the remaining Wave 4 interviews (*n* = 3) and two of the three pediatric participants, with available data, utilized the recall period as intended when responding. In any instance where a pediatric participant failed to apply the recall period appropriately when answering an item, the issue appeared to be more of an attention concern (i.e. remembering to apply the recall period to every item) rather than a comprehension issue, given that these same participants demonstrated a clear understanding of the recall period. Most pediatric patients indicated a preference for “past 24 h” versus “past 7 days”; however, preference results were mixed for caregivers. The final P-OMAQ-P and P-OMAQ-C include “past 24 h” and “past 7 days” versions.

##### Item content revisions

The following revisions were made to the P-OMAQ-P and the P-OMAQ-C items based on results from CD interviews: (1) the draft items to assess “taste of medicine” were revised to assess “taste of medicine before swallowing” to better distinguish it from the “aftertaste” items, (2) the “bother” items were removed because participants defined bother inconsistently (example definitions include feeling “frustrated” or “annoyed” by, getting “sick” from, or having a difficult time taking medicine), and (3) the “number of times taken daily” items were revised to align with the final recall periods (e.g. revised to “number of times you had to take the medicine”). Although dosing frequency was not spontaneously reported with a high frequency during the patient CE interviews (and thus was not reflected as a key attribute in Fig. 3), it was retained in the questionnaire based on results from feedback gathered during the CD component of the interviews. In addition, questions included in the draft P-OMAQ to assess overall happiness with/acceptability of the medicine, how much the medicine helps, the importance of taking it, willingness to keep taking it, and compliance (how often the child takes the medicine exactly as instructed) were retained based on feedback from respondents during the CD component of the interviews.

##### Response option continuum

Consideration was given during the development of the P-OMAQ to using a response option structure that would minimize the cognitive burden (i.e. reading burden) for younger patients; thus, the draft P-OMAQ-P and P-OMAQ-C were developed using a NRS. Two versions, a 5-point NRS and an 11-point NRS, were tested in Waves 1–3, and results found that the majority of pediatric (*n* = 11/19, 57.9%) and caregiver (*n* = 32/35, 91.4%) participants preferred the 5-point NRS over the 11-point NRS because it was easier to use. The final P-OMAQ uses a 5-point NRS.

##### “Happy” versus “acceptable”

Consideration was given during the development of the draft P-OMAQ to using a term in the item stem that would reflect “acceptable” in a way that could be understood by pediatric participants as young as 8 years of age. Therefore, the term “happy” was used in the P-OMAQ-P. The term “happy” was also used in the item stem for the P-OMAQ-C, for consistency with the pediatric questionnaire version. However, the draft P-OMAQ-C included questions to assess “overall acceptability” of the oral medicine, testing either “acceptable” or “happy” in the stem. Results were mixed with regard to caregiver interpretation and feedback relating to use of the term “acceptable” versus “happy”. Some caregiver participants interpreted these terms to mean the same thing, whereas others did not; no clear preference emerged.

##### Final content-valid P-OMAQ-P and P-OMAQ-C questionnaires

Items measured in the P-OMAQ-P and P-OMAQ-C are summarized in Table 1. The finalized P-OMAQ-P is a 12-item PRO for pediatric respondents of 8–17 years of age. The questionnaire is available with “past 24 hours” and “past 7 days” recall versions, and all items use a 5-point NRS with higher item-scores reflecting greater oral treatment acceptability. Seven items measure, for the oral medication taken in the “past 24 h” or “past 7 days”, how happy/unhappy the child is with the following: number of times taken daily; size or amount; smell; taste in the mouth before swallowing; taste in the mouth after taking the medicine; how the medicine feels in the mouth; and ease of swallowing. Four items measure overall happiness with the medicine, how much the medicine helps, the importance of taking the medicine, and willingness to keep taking the medicine. One item measures how often the child takes the medicine exactly as instructed.

The finalized P-OMAQ-C is a 19-item COA for adult caregivers (aged ≥18 years) of pediatric patients aged 6 months to 17 years. The questionnaire is available with “past 24 hours” and “past 7 day” recall versions, and all items use a 5-point NRS with higher item-scores reflecting greater oral treatment acceptability. Three PRO items measure, for the oral medication taken by the child in the “past 24 h” or “past 7 days”, how happy/unhappy the caregiver is with the following: number of times taken daily, size or amount, and smell. Five PRO items measured overall acceptability of the child’s medicine to the caregiver, happiness with the child’s medicine, how much the medicine helps the child, the importance of the child taking the medicine, and willingness to have the child continue to use the medicine. Eleven caregiver-reported outcome items measure, for the oral medications taken by the child now, what the caregiver has directly observed (or what the child has said) about the child’s experience taking the medicine. Of these, six items measured (based on the caregiver’s observation) how happy/unhappy the child is with number of times taken daily, size or amount, smell, taste, taste in the mouth after taking the medicine, and how the medicine feels in the mouth; one item measured the caregiver’s observation of the ease with which the child swallows the medicine; three items measured the caregiver’s perspective on overall acceptability to the child, happiness with the medicine, and willingness to continue to use the medicine; and one item measured the caregiver’s perspective on how often the child takes the medicine as instructed.

## Discussion

Although there is a need for routine assessment of pediatric acceptability throughout pharmacological and clinical development, no standardized measurement methods or evaluation criteria exist to define acceptability of pediatric medicines [9, 20, 22]. A recent study highlighted that, although there are several reports on pediatric acceptability, certain attributes are inadequately researched, including the effects that the shape, size, and dimension of tablets as well as the volume of liquids can have on acceptability [9]. This lack of knowledge prevents regulators from being able to issue robust guidelines on formulations for pediatric use [6]. Investigators are therefore unable to support pediatric formulation development adequately [57], and, consequently, no standardized rules or evidence for pediatric acceptability can be developed. The present study addresses the need for standardized COAs to assess key acceptability attributes for targeted oral formulations by conducting a concept-focused literature review and combining its results with opinions from clinical advisors and feedback from patients and their caregivers to develop standardized, content-valid questionnaires, named P-OMAQ, to assess pediatric medicine acceptability from the patient’s (P-OMAQ-P) and caregiver’s (P-OMAQ-C) perspectives.

Six key acceptability attributes emerged based on converging evidence from a literature review, panel discussions with clinical and scientific experts, and patient and caregiver interviews, and included: taste, aftertaste, swallowability, mouth feel, size or amount, and smell. These findings are consistent with another recently conducted literature review of pediatric oral medicine acceptability, which identified the following attributes: size and texture; shape; smell; taste, mouth feel, and aftertaste; number of units per dose/dose volume; measuring device; time and effort required to chew; time needed to dissolve/disperse; need for a measuring device; and ease of preparation [9].

Although the current study’s COA instrument-focused literature review identified nine PRO questionnaires that claimed to measure the acceptability of oral medicines in children, none of them adequately measured the comprehensive set of attributes identified and confirmed as important by patients and their caregivers (i.e. the existing PRO measures evaluated only some of the key attributes of acceptability).

Questions to assess the six key attributes identified were included in the final P-OMAQ, as well as additional items measuring dosing frequency, overall happiness with/acceptability of the medicine, how much the medicine helps, the importance of taking the medicine, willingness to keep taking the medicine, and compliance (how often the child takes the medicine exactly as instructed). These additional items were included for various reasons (e.g. the overall acceptability item was included to capture the respondent’s global evaluation of acceptability; the “how much the medicine helps” item was to capture the respondent’s perspective on efficacy; etc.). Certain attributes reported by participants were not included in the questionnaire because they were considered less critical for measurement (e.g. owing to a low frequency of reporting) or would likely be measured by other means in a clinical trial (e.g. side effects).

Results from the CD interviews demonstrated that pediatric participants and their caregivers were able to understand and respond to the final P-OMAQ items. Revisions were made throughout the waves of interviews that resulted in content revisions to the “number of times” item to align with the recall period; content revisions to distinguish “taste before swallowing” from “taste after swallowing”; a decision to drop the “bother” items (because the term “bother” was inconsistently interpreted); and 24-h and 7-week recall versions (with the recall period stated in the instructions and in each item). In the few instances where a pediatric participant failed to apply the recall period appropriately, the issue appeared to be more of an attention concern (i.e. remembering to apply the recall period to every item) rather than a comprehension issue. Administration of training prior to completion of the questionnaire may address this issue.

A recent study recommends hedonic scales as the first-choice tool in the assessment of the taste of a pediatric medicine [58]. The current research tested both a 5- and an 11-point NRS, and both were well understood by the pediatric participants and their caregivers. This scale structure was chosen to avoid using a verbal rating scale, as there was a concern that this would be difficult for children to understand. Overall, pediatric participants and their caregivers preferred the 5-point NRS, which was implemented as the response scale for the questionnaires based on these results.

The data gathered during these research activities resulted in the development of the P-OMAQ-P, a PRO for self-report by pediatric patients aged 8–17 years, and the P-OMAQ-C, a caregiver questionnaire that contains PRO items to assess the acceptability to the caregiver of the child’s medicine and observer-reported items to assess what the caregiver has directly observed (or what the child has said) about child’s experience of taking the medicine. The questionnaires are brief yet comprehensive, understandable, and easy to complete, creating minimal burden for their respective respondents. The P-OMAQ is the first empirically derived, content-valid COA questionnaire designed to measure and quantify acceptability of targeted pediatric oral medicines.

There are a few limitations of note with the current research undertaken to develop the P-OMAQ questionnaires. First, this research focused on the following oral formulations: mini-tablets and tablets, oral liquids or sprays, and powders or granules for reconstitution. These categories were chosen in order to be as inclusive and applicable as possible. Acceptability attributes measured in the P-OMAQ may not apply to more specific oral formulations (e.g. lozenges) or injectable formulations; however, the tool can serve as a starting point for alternative questionnaires. Second, the patient and caregiver interviews were carried out in a US population only, and the attributes of acceptability that are important to this cohort may differ from those that are important to other patient populations from different countries. Therefore, further CE and CD analyses should be performed in other countries to determine whether the questionnaires will be applicable in different geographies, cultures, and healthcare systems. Third, saturation of concept analyses was not conducted as part of this research. In a study by Turner-Bowker et al. (2018), conducted to evaluate appropriate a priori sample sizes for concept elicitation studies supporting questionnaire development, findings indicated that 99% of concepts emerged by the completion of the 25th interview [59]. Based on these findings, the number of participants interviewed in the current study should be sufficient to achieve saturation of concept. However, future qualitative research in other subgroups should evaluate saturation. Another limitation is that no data currently exist to document the dimensionality and scoring approach, nor the psychometric performance of the scores from the questionnaires; however, this research is planned for the future. Further quantitative research can also evaluate acceptability results by participant subgroups (e.g. based on condition type).

The P-OMAQ questionnaires are not yet final. Although content validity has been established, research is needed to confirm a scoring approach and to evaluate score psychometric performance. Quantitative psychometric analyses can also answer open research questions relating to the P-OMAQ. For example, results for testing either “acceptable” or “happy” in the stem of the draft POMAQ-C were mixed with regard to caregivers’ interpretation and feedback. Future analyses can evaluate possible redundancy of the alternative items that measure “acceptable” versus “happy”. Score interpretation guidelines must also be developed and produced prior to finalization and widespread use of the questionnaires in future pediatric clinical trials.

## Conclusions

Despite the importance of assessment of the acceptability of a medicine when developing therapeutic agents, there are currently no standardized COAs for comprehensively investigating medicine acceptability in pediatric populations. The present study addressed this unmet need by developing new content-valid patient and caregiver acceptability questionnaires, the P-OMAQ-P and the P-OMAQ-C, for the assessment of oral formulation acceptability. These questionnaires can be used in clinical trials to assess the acceptability of specific oral formulation types and can yield useful information that may facilitate pediatric drug development, approval, and marketing initiatives.

## Supplementary information

**Additional file 1: Table S1.** Search strategy for the conceptual literature review.

**Additional file 2: Table S2.** Participant recruitment by site and wave for the patient and caregiver interviews.

**Additional file 3:** Inclusion and exclusion criteria for the pediatric patient and caregiver interviews.

**Additional file 4: Fig. S1.** Process for identifying articles for the conceptual literature review.

**Additional file 5: Fig. S2.** Process for identifying articles for the literature review to identify existing acceptability questionnaires.

**Additional file 6: Table S3.** Additional caregiver demographic information.

**Additional file 7: Table S4.** Frequency of each acceptability attribute reported by pediatric participants during the concept elicitation interviews.

## Data Availability

The datasets generated and/or analyzed during the current study are available from the corresponding author on reasonable request.

## Abbreviations

CD: Cognitive debriefing
CE: Concept elicitation
COA: Clinical outcome assessment
EMA: European Medicines Agency
NRS: Numerical rating scale
P-OMAQ: Pediatric Oral Medicines Acceptability Questionnaires
P-OMAQ-C: Pediatric Oral Medicines Acceptability Questionnaires caregiver version
P-OMAQ-P: Pediatric Oral Medicines Acceptability Questionnaires patient version
PRO: Patient reported outcome
TFA: Theoretical framework of acceptability
