# Supplementary material for: Development and content validation of the Pediatric Oral Medicines Acceptability Questionnaires (P-OMAQ): patient-reported and caregiver-reported outcome measures
Source: J Patient Rep Outcomes. 2020 Oct 1;4:80. doi: 10.1186/s41687-020-00246-1 (PMC7527387; doi:10.1186/s41687-020-00246-1)
Supplement: Supplementary file 1 — Additional file 1: Table S1. Search strategy for the conceptual literature review. [file 41687_2020_246_MOESM1_ESM.docx]

# Additional file

Additional file 1: Table S1 Search strategy for the conceptual literature review

| Step | Search terms |
| --- | --- |
| 1 | oral AND (treatment OR medication OR formulation OR preparation) |
| 2 | powder OR granule |
| 3 | liquid AND (syringe OR spray OR solution OR reconstit ^a^ OR syrup OR mixture OR suspension OR drop) |
| 4 | tablet OR pill OR “coated tablet” OR ’mini tablet” OR “mini tablets” OR “small tablet” OR capsule OR (tablet AND (effervescent OR soluble OR dispersible OR chewable OR chewtab)) |
| 5 | (1) OR (2) OR (3) OR (4) |
| 6 | child ^a^ OR pediatric OR paediatric OR kid OR adolesc ^a^ OR teen OR infant OR baby |
| 7 | accept ^a^ OR palatab ^a^ OR smell OR taste OR aftertaste OR texture OR swallow ^a^ OR appear ^a^ OR color OR shape OR dose OR number OR frequency OR duration OR administration OR device OR discomfort OR pain OR packaging |
| 8 | (5) AND (6) AND (7) |
| 9 | Limit to children (0–18 years) |
| 10 | Limited to English language |
| 11 | Limited to 10 years (2007–2017) |
| 12 | Limited to peer-reviewed journal ^b^ |
| 13 | Limit to article type: journal (excludes conference abstracts) ^b^ |
| 14 | Duplicates removed – PRIMARY search results |

^a^ Indicates truncation used for search term

^b^Limit not valid in one or more databases

**Inclusion and exclusion criteria for the concept-focused literature review**

Abstracts were *included* for full-text review if they primarily focused on assessing the acceptability of oral formulation among children (aged ≤ 18 years). Abstracts were *excluded* if they were: not available in English; published 10 years before the date of the search; from nonpeer-reviewed publications; primarily focused on nonhuman research; and/or focused on non-oral medications
