# Supplementary material for: Development and content validation of the Pediatric Oral Medicines Acceptability Questionnaires (P-OMAQ): patient-reported and caregiver-reported outcome measures
Source: J Patient Rep Outcomes. 2020 Oct 1;4:80. doi: 10.1186/s41687-020-00246-1 (PMC7527387; doi:10.1186/s41687-020-00246-1)
Supplement: Supplementary file 2 — Additional file 2: Table S2. Participant recruitment by site and wave for the patient and caregiver interviews. [file 41687_2020_246_MOESM2_ESM.docx]

**Additional file 2: Table S2** Participant recruitment by site and interview wave for the patient and caregiver interviews

| Participants interviewed | Region 1  (New Orleans, LA, USA) | Region 2  (Chicago, IL, USA) | Region 3  (St Louis, MO, USA) | Total |
| --- | --- | --- | --- | --- |
| Number of clinical sites ^a^ | 1 | 15 | 3 | 19 |
| Wave 1 | 12 | 0 | 0 | 12 |
| Wave 2 | 0 | 7 | 6 | 13 |
| Wave 3 | 5 | 6 | 0 | 11 |
| Wave 4 ^b^ | 5 | 7 | 0 | 12 |
| Total | 22 | 20 | 6 | 48 |

^a^All clinicians recruited were pediatricians

^b^The goal of the Wave 4 interviews was to debrief cognitively the questionnaire among the youngest age group that would be asked to complete the questionnaire; therefore, only participants aged 8–11 years were asked to participate in this wave of interviews
