# Supplementary material for: Development and content validation of the Pediatric Oral Medicines Acceptability Questionnaires (P-OMAQ): patient-reported and caregiver-reported outcome measures
Source: J Patient Rep Outcomes. 2020 Oct 1;4:80. doi: 10.1186/s41687-020-00246-1 (PMC7527387; doi:10.1186/s41687-020-00246-1)
Supplement: Supplementary file 3 — Additional file 3: Inclusion and exclusion criteria for the pediatric patient and caregiver interviews. [file 41687_2020_246_MOESM3_ESM.docx]

**Additional file 3:** Inclusion and exclusion criteria for pediatric participant interviews

Participants were *included* if they met the following criteria.

- Caregiver/legal guardian has provided written informed consent for the minor to participate.
- Participant (aged 7–17 years) ^a^ has provided written assent to participate.
- Participant has a confirmed prescription for an oral medication of at least one of the following types:
  - powders or granules (for liquid reconstitution)
  - mini-tablets or tablets
  - oral liquid syringe or oral spray.
- Participant (aged 6–17 years) is fluent in English (i.e. able to speak, read, write, and comprehend) and is willing and able to complete study questionnaires and participate in a 30- to 90-minute face-to-face or telephone interview.

Participants were *excluded* if they met the following criterion.

- Participant has any condition or situation that would put the individual at significant risk, may confound the study results, or may interfere significantly with the individual’s involvement in the study, or participant has a cognitive impairment that would interfere with his/her participation in a 30- to 90-minute interview.

Caregivers were *include*d if they met the following criteria.

- Participant has provided written informed consent to participate.
- Participant provides daily care for a pediatric patient with a confirmed prescription for an oral medication of at least one of the following types:
  - powders or granules (for liquid reconstitution)
  - mini-tablets or tablets
  - oral liquid syringe or oral spray as provided by the clinician.
- Participant is fluent in English (i.e. able to speak, read, write, and comprehend) and is willing and able to complete study questionnaires and participate in a 30- to 90-minute, face-to-face or telephone interview.

Caregivers were *excluded* if they met the following criterion.

- Participant has any condition or situation that would put the individual at significant risk, may confound the study results, or may interfere significantly with the individual’s involvement in the study, or participant has a cognitive impairment that would interfere with his/her participation in a 30- to 90-minute interview.

^a^The assent form only captured written assent from children ages 7–17 years, which is why the 6-year-olds did not complete the assent form. This age threshold (7–17 years) was set by the Independent Review Board.
