# Supplementary material for: Development and content validation of the Pediatric Oral Medicines Acceptability Questionnaires (P-OMAQ): patient-reported and caregiver-reported outcome measures
Source: J Patient Rep Outcomes. 2020 Oct 1;4:80. doi: 10.1186/s41687-020-00246-1 (PMC7527387; doi:10.1186/s41687-020-00246-1)
Supplement: Supplementary file 4 — Additional file 4: Fig. S1. Process for identifying articles for the conceptual literature review. [file 41687_2020_246_MOESM4_ESM.docx]

Additional file 4: Fig. S1 Process for identifying articles for the conceptual literature review


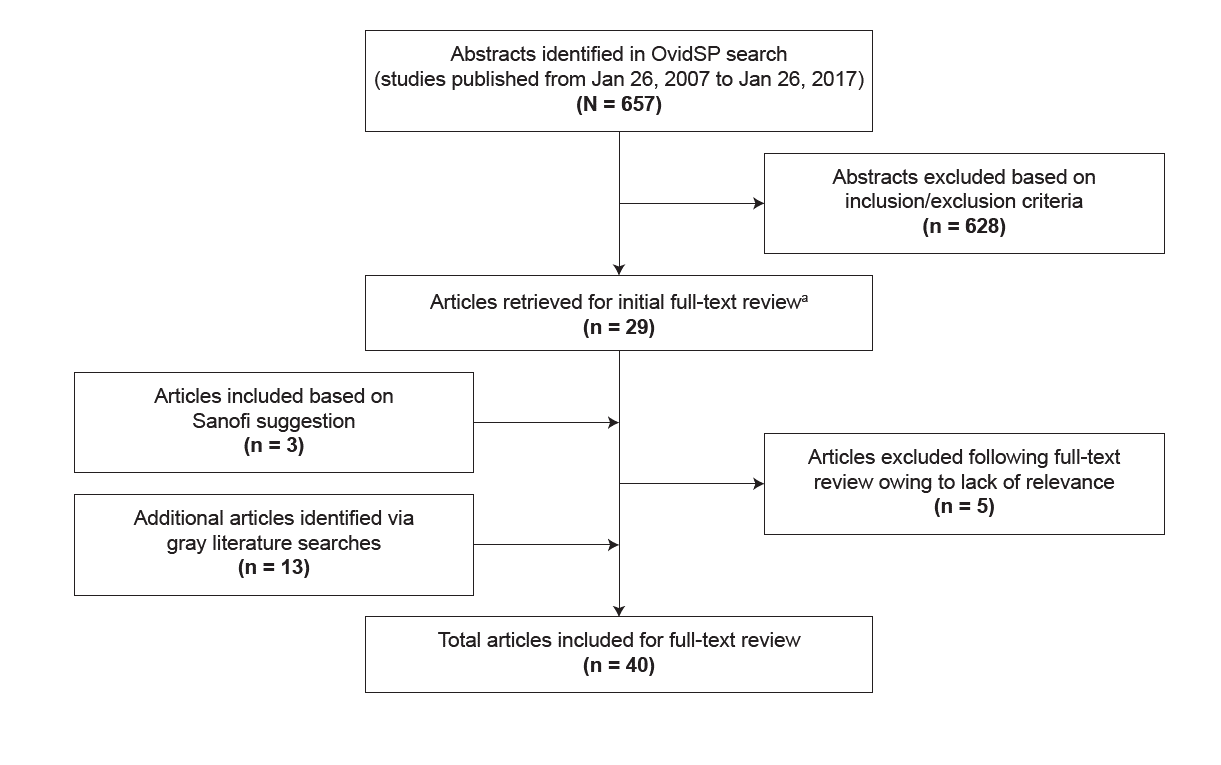


^a^Abstracts that best met the screening criteria and that presented the strongest likelihood of providing detailed information were selected for full-text review
