# Supplementary material for: Development and content validation of the Pediatric Oral Medicines Acceptability Questionnaires (P-OMAQ): patient-reported and caregiver-reported outcome measures
Source: J Patient Rep Outcomes. 2020 Oct 1;4:80. doi: 10.1186/s41687-020-00246-1 (PMC7527387; doi:10.1186/s41687-020-00246-1)
Supplement: Supplementary file 5 — Additional file 5: Fig. S2. Process for identifying articles for the literature review to identify existing acceptability questionnaires. [file 41687_2020_246_MOESM5_ESM.docx]

Additional file 5: Fig. S2 Process for identifying articles for the literature review to identify existing acceptability questionnaires


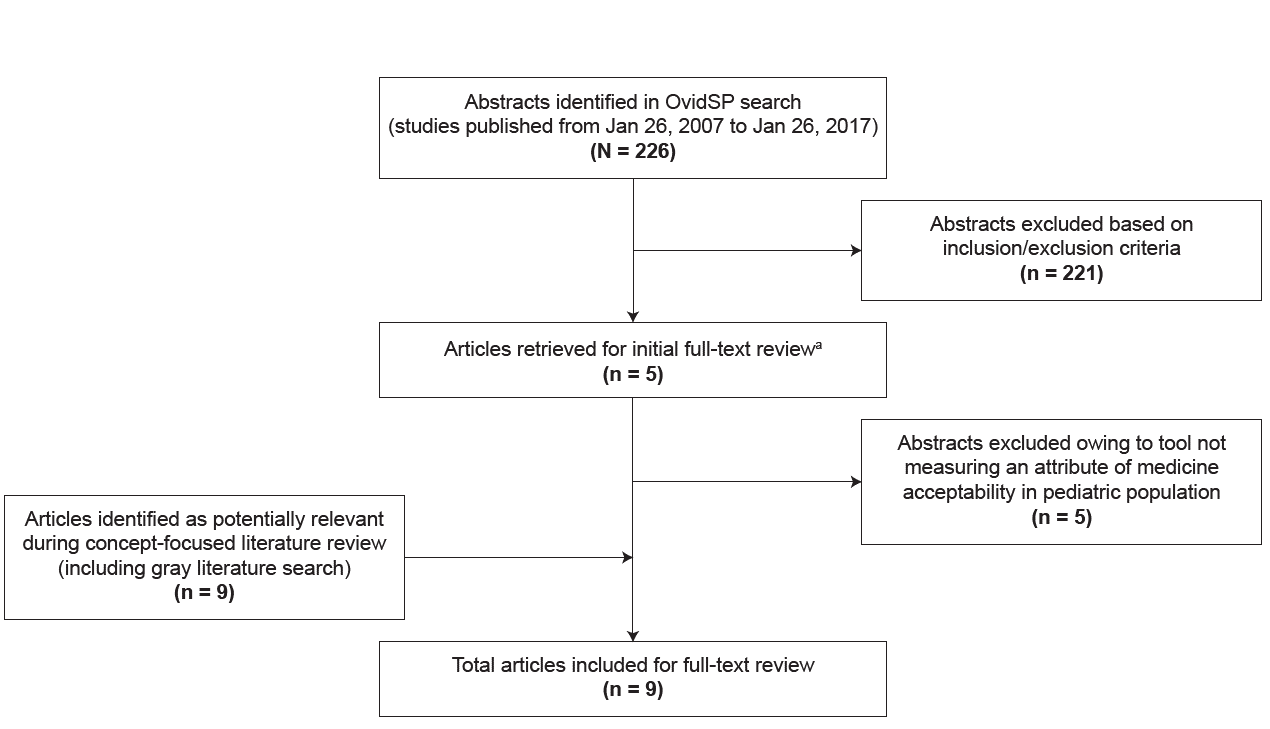


^a^Abstracts that best met the screening criteria and that presented the strongest likelihood of providing detailed information were selected for full-text review
