# Supplementary material for: Development and content validation of the Pediatric Oral Medicines Acceptability Questionnaires (P-OMAQ): patient-reported and caregiver-reported outcome measures
Source: J Patient Rep Outcomes. 2020 Oct 1;4:80. doi: 10.1186/s41687-020-00246-1 (PMC7527387; doi:10.1186/s41687-020-00246-1)
Supplement: Supplementary file 6 — Additional file 6: Table S3. Additional caregiver demographic information. [file 41687_2020_246_MOESM6_ESM.docx]

Additional file 6: Table S3 Additional caregiver demographic information

| Caregiver data | Total (*N* = 48) *n* (%) |
| --- | --- |
| Education | |
| High school diploma (or GED) or less | 5 (10.4%) |
| Some college or certificate program | 17 (35.4%) |
| College or university degree (two- or four-year) | 22 (45.8%) |
| Graduate degree | 3 (6.3%) |
| Annual household income | |
| Under $25,000 | 7 (14.6%) |
| $25,000 to $49,999 | 6 (12.5%) |
| $50,000 to $74,999 | 4 (8.3%) |
| $75,000 to $99,999 | 9 (18.8%) |
| $100,000 and over | 16 (33.3%) |
| Prefer not to answer | 6 (12.5%) |
| Work status | |
| Working full-time | 26 (54.2%) |

GED, General Educational Development
