# Supplementary material for: Development and content validation of the Pediatric Oral Medicines Acceptability Questionnaires (P-OMAQ): patient-reported and caregiver-reported outcome measures
Source: J Patient Rep Outcomes. 2020 Oct 1;4:80. doi: 10.1186/s41687-020-00246-1 (PMC7527387; doi:10.1186/s41687-020-00246-1)
Supplement: Supplementary file 7 — Additional file 7: Table S4. Frequency of each acceptability attribute reported by pediatric participants during the concept elicitation interviews. [file 41687_2020_246_MOESM7_ESM.docx]

**Additional file 7: Table S4** Frequency of each acceptability attribute reported by pediatric participants during the concept elicitation interviews

| Attribute | Formulation type | | | | Age | | | |
| --- | --- | --- | --- | --- | --- | --- | --- | --- |
|  | Frequency of participant report, *n*  (*N* = 36) | Tablet, *n*  (*N* = 12) | Powder, *n*  (*N* = 12) | Liquid, *n*  (*N* = 12) | 6 months to 5 years ^a^, *n*  (*N* = 13) | 6–7 years ^b^, *n* (*N* = 4) | 8–11 years ^b^, *n*  (*N* = 9) | 12–17 years ^c^, *n*  (*N* = 10) |
| Taste (taste before swallowing) | 30 | 11 | 8 | 12 ^d^ | 11 | 3 | 8 | 8 |
| Texture/ mouth feel | 30 | 9 | 10 ^d^ | 11 | 11 | 4 | 8 | 7 |
| Swallowability | 27 | 12 | 7 | 8 | 9 | 3 | 7 | 8 |
| Size/amount | 27 | 12 | 9 ^d^ | 5 | 10 | 2 | 8 | 7 |
| Aftertaste (taste after swallowing) | 17 | 4 | 7 ^d^ | 5 | 6 | 1 | 4 | 6 |
| Smell | 17 | 2 | 7 | 8 | 7 | 3 | 3 | 4 |
| Efficacy | 11 | 6 | 3 ^d^ | 2 | 4 | 1 | 2 | 4 |
| Dosing frequency | 3 | 2 | 0 | 1 | 1 | 0 | 2 | 0 |
| Side effects | 5 | 3 | 1 | 1 | 1 | 0 | 2 | 2 |
| Preparation | 12 | 2 | 4 | 6 | 10 | 0 | 1 | 1 |
| Appearance/ color | 8 | 1 | 3 | 4 | 4 | 0 | 1 | 3 |
| Overall ease of administration | 1 | 0 | 0 | 1 | 0 | 0 | 0 | 1 |

^a^Caregiver only

^b^Patient and caregiver

^c^Patient (caregivers were invited to participate); separate consent was obtained from the patient and caregiver

^d^The patient administered her/his medicine through a nebulizer
